# The indispensability of race in medicine

**DOI:** 10.1007/s11017-023-09622-6

**Published:** 2023-04-11

**Authors:** Ludovica Lorusso, Fabio Bacchini

**Affiliations:** 1 Department of Social Psychology, Universitat Autònoma de Barcelona, Barcelona, Spain; 2 Laboratory of Applied Epistemology, DADU, University of Sassari, Alghero, Italy

**Keywords:** Race, Race correction, Racism, Clinical medicine, Epidemiology, Fundamental causes

## Abstract

A movement asking to take race out of medicine is growing in the US. While we agree with the necessity to get rid of flawed assumptions about biological race that pervade automatic race correction in medical algorithms, we urge caution about insisting on a blanket eliminativism about race in medicine. If we look at racism as a fundamental cause, in the sense that this notion has been introduced in epidemiological studies by Bruce Link and Jo Phelan, we must conclude that race is indispensable to consider, investigate, and denounce the health effects of multilevel racism, and cannot be eliminated by addressing more specific risk factors in socially responsible epidemiology and clinical medicine. This does not mean that realism about human races is vindicated. While maintaining that there are no human races, we show how it is that a non-referring concept can nonetheless turn out indispensable for explaining real phenomena.

## The demand to take race out of medicine

Many scholars have proposed in the past years to eliminate all race-related terms from scientific and medical discourse to avoid the risk of genetic or molecular reification of racial categories and the perpetuation of the pernicious effects of racism [1]. For example, Yehudi Webster [2] has argued that races have no biological reality, and eliminating them from our discourses is not only ontologically serious but also an effective strategy to fight against racial discrimination. Naomi Zack [3, p. 307] has expressed scepticism about the capacity of race-terms to be successfully reconstructed into expressions with morally just meanings and has concluded that likely “a more direct […] route to freedom would be to simply let the whole idea of ‘race’ go;” and K. Anthony Appiah [4] has argued in the same spirit that the best move is to drop racial classifications completely. More recently, some scholars have urged geneticists to develop genetic research into complex diseases without recurring to the concept of race and using only race-free categories of genetic difference [5].

Today this debate is revived by an increasing attraction to the problem of race correction in medicine, i.e., the systematic race adjustment in algorithms and guidelines to determine death risk or necessity of medical prescription. For example, because Black patients are presumed to have higher muscle mass and a creatinine generation rate than patients of other races, their eGFR (estimated glomerular filtration rate, a value commonly used to measure the level of kidney function) is routinely multiplied by 1.16/1.21 more than the eGFR for White patients [6], which results in delayed diagnosis of kidney disease and transplant referral. Another racially discriminatory medical practice is that of measuring lung capacity using spirometers that are programmed to automatically apply a race-based correction factor to the reading, lowering a “normal” reading for a Black person by 10% to 15%. This evidently produces delay in care and inadequate treatment for Black people [7].

Vyas et al. [8] offer up to 13 examples of such widely used algorithms unfairly embedding race into decisions of health care and exhort clinicians to advocate for their institutions to remove the adjustment for race when inappropriate. Many providers, however, are starting to consider race correction as *always* inappropriate. While agreeing that medicine should move forward from “race-based” to “race-conscious” medicine [9], a growing number of thinkers today believe that the only coherent form of race-conscious medicine is a race-free medicine. As stated by Cerdeña and colleagues [9, p. 1125], “research in clinical medicine and epidemiology requires explicit hypotheses; however, hypotheses involving race are frequently implicit and circular, relying on conventional wisdom that Black and Brown people are genetically distinct from White people.” Indeed, Black Americans are also systematically undertreated for pain relative to White Americans due to the perpetuation of the racist belief, which dates back to slavery, that Black people are genetically more tolerant of pain [10].

A growing number of medical professionals are urging medical centres across the USA to take race out of health care at this very moment; the American Medical Association galvanised the movement by taking position in favour of “ending the practice of using race as a proxy for biology in medical education, research and clinical practice” and “acknowledging that race is a social construct and not an inherent risk factor for disease” [11] (see also Richter and Best [12]). A group of medical students and graduate student researchers of UC Berkeley wrote in 2020 a report titled “Toward the Abolition of Biological Race in Medicine” where they deplore to be “taught that race can serve as a risk factor for disease” and claim that “racial health disparities are often wrongly attributed to biology and physiology of racial groups rather than the stratified socioeconomic opportunities that are available” [13, p. 1].

Most of these reports and documents take side only against unquestioned and flawed assumptions about biological race that pervade medicine, not necessarily against all possible uses of race in medical thought. Still, the impetus of the campaign against the current misuse of race as a risk factor is so great that it might easily translate into the request to adopt the elimination of all reference to race in medicine as an antiracist institutional practice. There is an inherent ambiguity in the movement concerning this specific point. The UC Berkeley report, for example, is overtly in favour of a world “where the health consequences of racism are acknowledged, addressed, and cared for in all their forms,” but it lines up against “race-based medicine” because it “increases stress and the burden of racist stigma” in people of colour: “if we don’t dismantle race-based medicine, it will be perpetuated, ultimately harming patients in real, concrete ways” [13, p. 4]. Thus, on occasion, the report inadvertently makes statements that seem to take position for the elimination of the race category from clinical medicine. For example:

Race also tends to overwhelm the clinical measures. It blinds doctors to patients’ symptoms, family illnesses, their history, their own illnesses they might have—all more evidence-based than the patient’s race. Race can’t substitute for these important clinical measures without sacrificing patient well-being [13, pp. 27–28].

The authors of the report are resolutely in favour of the idea that race is not a biological concept, but rather a sociohistorical construct and concept [13, p. 28]. Moreover, they deplore that “we as providers not only fail to address in practice how racism is creating health disparities, we also create and perpetuate racial health disparities” [13, p. 7]. They fail to explicitly conclude, however, that developing “a framework for understanding racism and health disparities in medicine” [13, p. 6] requires an intense use of the aforementioned sociohistorical concept of race in medical thought. Rather, on occasion they seem to think that the plan of discussing racism in clinical health and the health sciences as a meaningful determinant of health outcomes is consistent with our stopping conceiving and using race (indeed, not just biological race, but race *tout court*) as a risk factor. They write:

Medicine must acknowledge that use of race as a risk factor or predictor of health outcomes is simply false science [13, p. 15].

The report commendably takes sides against “use of racial identities as sole determinants of health outcomes [that] frames Black people as having “inherently” poor cardiovascular health” [13, p. 21], but ends up opposing literally any employment of race as a proxy or variable for diagnostic purposes and treatment indications, when it concludes, for example, that “using race as a heuristic for diagnosis of disease and interpretation of symptoms masks racism” and that “if we don’t dismantle race-based medicine, it will be perpetuated” [13, p. 28].

The same ambiguity can be found in most of the other documents describing and supporting the movement. For example, in the Penn Medicine and the Afterlives of Slavery Project (PMAS) web page — devoted to investigating and combating automatic race corrections in medical diagnosis and treatment — it is said that “PMAS is committed to eliminating race-based medicine, especially the use of automatic race corrections, in medicine at Penn and around the world;” and the title of the page is “Eliminating race from medical practise,” without further specification [14].

The paper by Cerdeña, Plaisime, and Tsai [9] mentioned above, too, despite its repeated emphasis of the importance of a race-conscious approach, seems to recommend the banning of race in medical thought in deploring, for example, “on the wards, students learn that race is relevant to treatment decisions,” or when it says:

Even if significant findings or clinical anecdotes support the use of racially tailored practices, they should be rigorously critiqued and mediating variables, such as structural conditions, should be analysed accordingly [9, p. 1125].

While we resolutely agree with the movement about the urgent necessity to get rid of race correction from clinical practise, at the same time we urge caution about insisting on a blanket eliminavitism of race from medicine. We doubt that it would be a good idea to completely erase the race concept from science or medicine; in some epistemic contexts, race is simply indispensable as a category. This is very sad to say — since the reason of its in-eliminability is the persistence of the effects of past and present racism on health — but is no less true. While it is important to make a stand against uncritical and routine use of race, it is a mistake to oppose the use of race *tout court* if one’s aim is to highlight, study, and contrast the biological effects of racism and racial health disparities. Critical use of race is necessary.

We need to be very clear about one point. We are not arguing that indiscriminate eliminavitism of race is wrong *because* there are such things as human races. Quite the contrary, we acknowledge that, from a population genetics view, human races (plural) do not exist, in the sense that one cannot non-arbitrarily classify humankind into discrete genetic clusters or populations constructed on the basis of some kind of genetic variation [15–21]. Even if one professes moderate realism, or conventionalism, about “bio-genomic clusters” as obtained by Rosenberg et al. [22] through a software like STRUCTURE, which investigates population structure by using multi-locus genotype data, we agree with Kaplan and Winther [18] that one should be definitely antirealist about biological races, when the issue at stake is whether there is a stable mapping between the social groups identified as races and *one special* particular set of bio-genomic clusters. This is because there is an endless number of unmatching sets of bio-genomic clusters, each set being the result of one very specific package of highly arbitrary choices, and all being of the same rank with regard to their ontological status [16, 21].

Indeed, the majority of the philosophers of biology would promptly agree that there are no *genetic* or *biological* races but would at the same time concede that some other kind of races exist — typically *social* races [23, 24]. This result is often obtained by distinguishing between a biological (or, genetic) and a social concept of race and concluding that, while the former does not refer, the latter does [18, 19]. We prefer a more straightforward approach [21]. After all, since it was decided that there is no such a thing as witchcraft, the claim has *not* wavered, *nor* has it been suggested that, while the concept of demonic witchcraft does not refer, some social concept of witchcraft does. Quite the contrary, the judgement that there is no witchcraft has been uncomplicatedly held — even acknowledging that witch hunts take place even today (for example, in Tanzania about 40,000 people were accused of witchcraft and murdered still between 1960 and 2000) [25].

But even if there are no such things as human races, our position is that some real phenomena — and even some real *biological* phenomena — cannot be adequately explained without resorting to the concept of race. So, we aim to show in what fields of medical sciences the concept of race is (at least today) ineliminable, despite its not referring, contrary to the position expressed by many scholars who have presupposed that, if there is no such a thing as race, it obviously must be eliminated from *medical* discourse.

## Why race should *not* be eliminated from all medical discourse

Apparently, there is nothing more irreproachable than eliminating a non-referring concept from scientific discourse. But this is a mistake. Indeed, on occasion, one cannot adequately explain a real phenomenon without recurring to a non-referring concept. Take, again, the witchcraft example. How can one *explain* what happened to an estimated 50,000 people who were burnt at the stake in Europe from 1580 to 1630 unless one makes reference to the concept of witchcraft? Sure, the concept was causally active not because it referred, but because it occurred in propositional attitudes in many people’s minds that translated into physical actions on their part. Nonetheless, the concept had a key causal role in producing a large number of *biological effects* on human bodies such as injuries, burning, and death.

The same can be said about race. Race is a non-referring concept that relevantly appears in several beliefs and desires, driving people to act in one way rather than in another towards each other. All these actions, which would not be performed otherwise, produce concrete biological effects on human bodies. Trying to explain these effects without the concept of race would be like trying to account for the thousands of people burnt in Early Modern Europe by appealing only to the concept of fire and pyre. The relevantly explanatory properties at issue here are the properties of being considered a witch and of being considered of a certain race, not directly the properties of being a witch and of being of a certain race (which are never instantiated). Still, the concepts of witchcraft and race are ineliminable from the explanation.

But what are exactly the phenomena that cannot be adequately explained without the concept of race, and which are the areas of medicine where the elimination of race would be harmful?

Our warning that race should not be eliminated from medical thinking relies on some important recent works which stress that race can be a medically useful category despite it not being a proxy for genetic differences [17, 26]. We agree with Gravlee [26, p. 53] that “the view of race as a cultural construct needs to become a starting point for empirical research, rather than an end point for the dismissal of race”—this empirical research aiming at identifying “the pathways of embodiment through which race become biology.”

While what Gravlee has in mind is the specific contribution of cultural anthropology to the unveiling of the origins and persistence of particular racial inequalities in health (see also the papers he refers to: [27–29]), we believe that a new comprehensive eco-social paradigm in epidemiology is needed — in the wake of what has been proposed by Krieger [30, 31] — in which the importance of the race variable is fully acknowledged by epidemiologists themselves and used systematically, not just on input from cultural anthropologists (but see, also, Dressler, Oths and Gravlee [32]).

Race will unfortunately continue to be a precious epidemiological variable, because it is simply indispensable for accounting for an important subset of patterns of unequal distribution of disease in the general population. So, we think that our discussion about the possibility of eliminating race from medicine should start from the epidemiological usefulness of race—a problem that is intertwined with the question of the opportunity for epidemiology to be constitutionally ethically involved in uncovering and contrasting health inequalities between populations — a matter more and more debated today both by epidemiologists and philosophers of epidemiology [33, 34].

This is not to say that the issue whether race-specific drugs or the use of the race category in clinical medicine are admissible (the only dimensions of the question about the legitimacy of a possible medical use of race in medicine taken into account by Kaplan [17])  should be set aside. But it seems to us that a preliminary reflection needs to be made about whether a medical use of race can be helpful to fight racism, and that such a reflection primarily entails discussing to what extent epidemiology should have the goal of addressing social determinants of health inequalities between populations, and what epistemological role the use of the race variable can play in achieving this aim.

Indeed, the epidemiology community has recently been home to intense debate over the necessity to become fully aware of its social responsibilities and to focus on strategies for denouncing and reducing health inequalities within and between populations [35, 36]. So, how can epidemiologists point the finger at the health consequences of racism and combat racial health inequities if they do not make use of the concept of race?

One could reply that the fact that the risk of morbidity and mortality from most complex or multifactorial disease is patterned along racial lines in all Western countries [37] can be explained by factors which overlap with race and are more directly responsible of what is observed, such as socioeconomic status (SES), environmental exposures, neighbourhood violence, food quality, access to health care, housing conditions, education, access to information, and so on. So, why should one not disaggregate race into these variables to analytically account for the mechanisms through which racism affects health and, at the same time, get rid of an ontologically discredited and socially harmful category like race?

The answer is twofold. First, should epidemiology focus exclusively on risk factors more proximate than race, it would give up the mission of denouncing the health inequities caused by multilevel racism, because epidemiological data would only reveal, for example, that people exposed to more air pollution are more at risk of developing many diseases such as asthma and dementia [38], but would be silent about how racism considerably increases black people’s fine particulate matter exposure via SES, residential segregation, environmental quality, and so on (see [39] for an appeal to epidemiologists to stop being “prisoners of the proximate”). Second, race does not simply reduce away like that. There is a correlation between race and health that remains even if the other variables as SES are controlled [40]. Thus, a race-free epidemiology would not just be reprehensibly blind to racism but also resign itself to an avoidable explanatory gap.

In 1995, Bruce G. Link and Jo Phelan introduced in epidemiological studies the fundamental cause theory, developed after Lieberson’s [41] concept of basic causes (see Clouston and Link [42] for a comprehensive and updated review). According to the theory,

[…] a fundamental social cause of health inequalities has four essential features. First, it influences multiple disease outcomes, meaning that it is not limited to only one or a few diseases or health problems. Second, it affects these disease outcomes through multiple risk factors. Third, it involves access to resources that can be used to avoid risks or to minimize the consequences of disease once it occurs. Finally, the association between a fundamental cause and health is reproduced over time via the replacement of intervening mechanisms [43, S29].

Link and Phelan [44] specifically argued that SES is a fundamental cause but introduced fundamental causality as a generic concept. The key idea is that a fundamental cause affects health through a myriad of causal mechanisms that are continuously renewed over time; also by virtue of the fact that the fundamental cause involves access to flexible resources that considerably influence a given outcome. As a result, the relation between the fundamental cause and the outcome remains also if one controls for a large number of risk factors related to the intervening mechanisms. Moreover, this relation survives through social change. They write:

New circumstances may arise that diminish or eliminate particular pathways connecting the fundamental cause to the outcome. However, when new circumstances that affect the outcome emerge or are created, the superior resources of some groups will tend to advantage them in terms of the new circumstance and will tend to create a new pathway connecting the fundamental cause to the outcome. The ebb and flow of specific pathways and the replacement of old pathways with new pathways connecting the fundamental cause to the outcome result in an enduring connection between the fundamental cause and the outcome [45, p. 314].

When there are fundamental-cause processes in play, causal mechanisms are multiple and replaceable, and undesirable outcomes cannot be combatted just by addressing intervening mechanisms. Nor it is possible to eliminate the fundamental cause by considering only the risk factors related to the intervening mechanisms. The problem is twofold: synchronic irreducibility and rapid ageing of relevant knowledge.

Phelan and Link [45] propose that racism is a fundamental cause of health inequalities. They argue that racism is a fundamental cause of SES (which as we have said is a fundamental cause of health inequalities); but racism is also a fundamental cause of health inequalities in its own right and independently of SES. Their argument in support of the latter statement consists in showing that racism satisfies all of the four defining properties of a fundamental cause: first, race is related to multiple disease outcomes [37]; second, it is so related via a plethora of mechanisms independent of SES (e.g., social stress, which can be divided into experiences of discrimination, other stressors like traumatic events, and consequent allostatic load, which is defined as “the ‘wear and tear’ the body experiences when repeated allostatic responses are activated during stressful situations” [46, 47], lower-quality medical care [48, 49], neighbourhood segregation effects [which can be distinguished into less recreational resources, bad effects on nutrition, increased probability to develop harmful substances addiction, poorer police protection and higher crime rates] [50, 51], and so on); third, there is a set of flexible race-related resources independent of SES (nonoccupational prestige and power [52, 53], beneficial social connections related to neighbourhood segregation [54, 55], and freedom - intended as the ability to control one’s own life circumstances and actions in Sen’s [56] sense [57–59]) - that avoid risks or minimize the consequences of disease; and fourth, racial inequalities in health are reproduced over time via the replacement of intervening mechanisms independent of SES [60–62].

If one agrees with Phelan and Link that racism is a fundamental cause of significant health inequalities (see Laster Pirtle [63] for the proposal that “racial capitalism” can be considered as a further and distinct fundamental cause of COVID-19 pandemic inequities in the United States), it follows that the only way for epidemiology to be socially responsible is to make use of the category of self- and other-identified race. Consider that, when one say that SES is a fundamental cause of health inequalities, one should more precisely refer to the different distribution of SES in the population — for, if all the individuals in the population had the same SES, SES could evidently cause no health *difference*, and *a fortiori* no health inequality (being at best a *cause of incidence* in Geoffrey Rose’s [64] sense). In a like manner, racism can only be a fundamental cause of health *differences* if one assumes a heterogeneity of exposure across the population (i.e., a population of racists all identified as belonging to the same race being a population where racism, although maybe affecting everybody’s health, is incapable of producing health differences). The point is that because heterogeneity of exposure to racism requires heterogeneity in one’s perceived race, self- and other-identified race is an indispensable variable to address racism as a fundamental cause of health inequalities.

The consequence of all this is that one cannot eliminate race from epidemiology. While one should actively fight against considering race as an essential, genetic variable, one should at the same time acknowledge that it captures the biological effects of racism unlike any other variable, and, consequently, one cannot expel it from epidemiology with a light heart. When the American Medical Association [65] declares to “support the creation of external policy to combat racism and its effects and encourage federal agencies and other organizations to expand research funding into the epidemiology of risks and damages related to racism,” it must be clear that this can only be done by using the race variable. Paradoxically, the more intensively and critically one employs race today, the earlier one will be able to get rid of it in the future. Race is essential for achieving the goal of denouncing the extent of the influence of racism on human biology. It would be just self-defeating for an anti-racist epidemiology to get rid of race at the present time.

In like manner, one should not presume that clinical medicine can do without race. For again, only other- or self-identified race grants clinicians epistemic access to the aetiology of the disease inasmuch as it depends on experienced racism or the epigenetic effects of racism as experienced by one’s ancestors [66–69]. Certainly, one must eliminate race from clinical settings in all those circumstances in which its use is only functional to reinforce health inequities and is grounded on false ideas about genetic and even hierarchical differences among human groups — as it is the case in most, if not all, race-adjusting clinical care guidelines used and recommended so far. But a precision medicine strategy requires considering self- and other-identified race, simply because there is no available set of biomarkers that can compete with race in terms of the capacity to capture the cumulative effects of multilevel racism on individual biology. As said, racism is a fundamental cause of health differences; intervening mechanisms are several and replaceable, and related risk factors are multiple and ephemeral. Just as one cannot eliminate race from epidemiology by just focusing on distal social factors of risk, one cannot eliminate race from clinical medicine by addressing biological factors increasingly proximate to disease. Race does not just reduce away.

Take the example of preterm birth. Preterm birth is 58,2% more likely to occur in Non-Hispanic Black Women than it is in Non-Hispanic White Women in contemporary U.S. [70]. Babies born prior to 37 weeks’ gestation are at increased risk for neonatal morbidity and mortality; preterm birth is the direct cause of 35% of all neonatal deaths worldwide; and survivors remain at high risk for complications in early childhood, adolescence, and into adulthood [71, 72]. Preterm birth is a complex phenotype that can hardly be explained by a single gene or a single environmental exposure. Racial differences in prematurity risk are the typical differences that are very likely to have racism among their fundamental causes [73]. Thus, it would be simply unacceptable for American obstetricians and gynecologists not to consider the patient’s race when estimating prematurity risk and making related important health decisions.

As for race-specific drugs, one should not ignore that some particular drugs might have systematically different effects in Black and White patients in a particular society, not because there are racial genetic differences affecting the quantifiable change in disease processes that result from the pharmacological or physical properties of the active treatment, but just because the causal pathways producing disease states in Black and White patients are relevantly different [17].

It is possible that a social cost will be paid from continuing to use race in health care. For example, the idea of race as a biological category that naturally produces health disparities because of genetic difference could be reinforced in students of medicine and public health, trainees, clinicians, and providers. The ongoing use of race in medical discourse might strengthen the racialization of society and the belief that racial classification is “natural” and inexorable. Moreover, people might develop resigned attitudes towards their medical conditions or classes of risks, and this could in turn negatively affect patient compliance and the effort to live a healthy lifestyle [74]. But the total social cost of eliminating race would probably be even higher because it would not only preempt acceptable epistemic access to the individual biological consequences of racism, but would also involve becoming blind to health inequalities produced by systemic racism. In this sense, critically employing race in clinical medicine might serve to combat “outdated, oppressive “normal ways of doing medicine” that have exploited Black and brown bodies” and “advance an antiracist, people-centered medicine” [13, p. 2]. This means that, should one decide to approve a race-specific drug, for example, one must clearly communicate that it is not that the drug has systematically different effects in Black and White people because there are some racial disease-related genetic differences, but that the drug has a higher success rate with specific disease aetiologies reliably associated with social determinants of health (i.e., the ongoing effects of perceived racism that are more likely to be found in Black than in White people). This could help people understand that the “real treatment” is the systematic fight against racist behaviour in our society (in this regard see Krimsky [75] on the instructive history of BiDil, the first race-specific drug approved by the U.S. Food and Drug Administration in 2005).

## Conclusion

Whatever decision one takes concerning race in clinical medicine, there is no good reason to continue employing race in the field of medical genetics, where it is currently used as a non-intrusive proxy for human continental populations tracking genetic ancestry (which in turn are thought to be good proxies for medically relevant genetic traits). In fact, first, self- and other-identified race turns out to be not a good proxy for continental population primary membership, and second, there is no certainty that continental population primary membership is a good proxy for genetic variation contributing to common complex diseases (so, at best, genetic ancestry — though not race — can be a proxy for rare genetic disorders) [21, 74]. But as Yudell et al. [76, p. 565] concede while advocating for phasing out all racial terminology in medical genetics and even in biological sciences,

Using race as a political or social category to study racism and its biological effects, although fraught with challenges, remains necessary. Such research is important to understand how structural inequities and discrimination produce health disparities in socioculturally defined groups.

In sum, indiscriminate eliminivatism of race in medicine is a mistake. As long as the biological effects of racism are present in our societies, the race variable remains epistemically necessary in many areas of medicine.
